# Frailty and chronic kidney disease: associations and implications

**DOI:** 10.1590/2175-8239-JBN-2022-0117en

**Published:** 2023-04-17

**Authors:** Luv Bansal, Ashish Goel, Amitesh Agarwal, Rahul Sharma, Rajarshi Kar, Alpana Raizada, Rhea Wason, Raghav Gera

**Affiliations:** 1 Max Super Speciality Hospital Vaishali, Ghaziabad, India.; 2 Dr. B.R. Ambedkar State Institute of Medical Sciences, Department of Medicine, Sahibzada Ajit Singh Nagar, India.; 3 University College of Medical Sciences, Department of Medicine, New Delhi, India.; 4 University College of Medical Sciences, Department of Community Medicine, New Delhi, India.; 5 University College of Medical Sciences, Department of Biochemistry, New Delhi, India.; 6 Maulana Azad Medical College, New Delhi, India.

**Keywords:** Renal Insufficiency, Chronic, Frailty, Depression, Glomerular Filtration Rate, Insuficiência Renal, Crônica, Fragilidade, Depressão, Taxa de Filtração Glomerular

## Abstract

**Introduction:**

Frailty and its association with chronic kidney disease (CKD) has been established previously. The present study examined this association further by studying the distribution of frailty among groups defined by different stages of the disease. It also identified associated health deficits and explored their association with estimated glomerular filtration rate (eGFR) and urine albumin creatinine ratio (UACR).

**Methods:**

A cross-sectional survey was conducted on 90 non-dialysis dependent CKD Stage 1–4 patients, recruited in three stratified groups of 30 participants each based on the stage of disease. Frailty was assessed using Fried’s frailty criteria and associated health deficits were recorded using a pre-determined list. Depression was screened using a 4-point depression scale.

**Results:**

21.1% of the participants were frail and 43.3% were pre-frail. The proportion of frailty in CKD groups A (Stages 1 and 2), B (Stage 3a), and C (Stages 3b and 4) was 10%, 13.3%, and 40%, respectively. The association of health deficits including co-morbidities, physical parameters, mental status, daily activities, etc. with UACR, eGFR, and CKD stages was not statistically significant. Nearly one in two frail participants was depressed compared with 14% among non-frail participants.

**Conclusion:**

The skewed distribution of 21% frail subjects identified in our study indicates an association between frailty and advancing kidney disease. Frail individuals had a lower eGFR, higher UACR, were more likely to be depressed, and had higher count of health deficits and poorer performance on Barthel Index of Activities of Daily Living and WHOQOL. Early identification of depression would improve care in these patients.

## RESUMO

**Introdução:**

Fragilidade e sua associação com DRC foram estabelecidas anteriormente. O presente estudo aprofundou esta associação, estudando distribuição da fragilidade entre grupos definidos por diferentes estágios da doença. Também identificou déficits de saúde associados e explorou sua associação com taxa de filtração glomerular estimada (TFGe) e relação albumina/creatinina urinária (RAC).

**Métodos:**

Realizou-se uma pesquisa transversal em 90 pacientes com DRC Estágios 1–4 não dependentes de diálise, recrutados em três grupos estratificados de 30 participantes cada, conforme estágio da doença. Avaliou-se fragilidade usando os critérios de fragilidade de Fried e registraram-se os déficits de saúde associados usando uma lista pré-determinada. A depressão foi verificada utilizando a escala de depressão de 4 pontos.

**Resultados:**

21,1% dos participantes eram frágeis e 43,3% eram pré-frágeis. A proporção de fragilidade nos grupos de DRC A (Estágios 1 e 2), B (Estágio 3a), e C (Estágios 3b e 4) foi de 10%, 13,3%, 40% respectivamente. A associação de déficits de saúde, incluindo comorbidades, parâmetros físicos, estado mental, atividades diárias etc. com RAC, TFGe e estágios da DRC não foi estatisticamente significativa. Cerca de um em cada dois participantes frágeis estava depressivo comparados com 14% entre não frágeis.

**Conclusão:**

A distribuição enviesada de 21% dos indivíduos frágeis identificados em nosso estudo indica associação entre fragilidade e doença renal progressiva. Indivíduos frágeis apresentaram menor TFGe, maior RAC, eram mais propensos a depressão, tinham maior índice de déficits de saúde e desempenho inferior no Índice de Atividades da Vida Diária de Barthel e WHOQOL. A identificação precoce da depressão melhoraria o atendimento desses pacientes.

## Introduction

Frailty, a well-established biologic syndrome of decreased reserve and resistance to stressors, resulting from cumulative declines across multiple physiologic systems, causes increased vulnerability to adverse outcomes. A systematic review revealed that the prevalence of frailty ranged from 7% among the community dwellers with chronic kidney disease (CKD) (stage 1–4) to 73% in a cohort of patients undergoing haemodialysis^1^. The incidence of frailty was found to increase with reducing glomerular filtration rate (GFR). The overall prevalence of frailty in patients suffering from end stage renal disease undergoing hemodialysis was found to be 46%^2^. Over 40% of these patients are under 40 years of age and over three quarters are over 60 years of age. Frail individuals account for 42% of those on regular hemodialysis^3^. Of these, 35% of younger patients and 50% of older ones are frail.

Frailty has been recognized as an aggregate of multiple small molecular-level injuries, which can be genetic, environmental, or random, and manifest over time as decreased resilience, reduced adaptability, and impaired homeostasis. CKD itself, is a state of accelerated metabolic aging, associated with protein-energy wasting, anemia, chronic inflammation, acidosis, hormonal disturbances, oxidative stress, insulin resistance, vascular calcification, osteoporosis, and accumulation of advanced glycation end-products^4^. Their association probably makes the injury worse resulting in further and accelerated damage. Due to this impaired homeostasis, frailty may be associated with loss of muscle mass and mortality, in addition to a higher risk of falls, functional decline, hospitalization, and institutionalization.

Among National Health and Nutrition Examination Survey (NHANES) participants aged 20 to 81 years, all stages of CKD, including microalbuminuria with preserved kidney function (estimated glomerular filtration rate [eGFR] > 60 mL/min/1.73 m^2^), were associated with significantly higher odds of frailty compared with individuals without CKD^5^. A higher risk of hospitalization and death has been found to be associated with frailty in incident dialysis patients. Also, frail patients start dialysis at a higher eGFR on average than non-frail patients. If frailty was secondary to uremia, initiation of dialysis would improve frailty in such patients. Loss of muscle mass, which is central to the construct of frailty, leads to lower creatinine generation with resultant overestimation of eGFR by creatinine-based equations, leading to higher eGFR at dialysis initiation. If frailty in these patients were secondary to uremia, it would improve upon initiation of dialysis. However, it appears that dependence in activities of daily living (ADLs) increases after dialysis initiation^6^.

Several studies demonstrated that frailty could be reversed or attenuated by interventions such as physical exercise and rehabilitation, nutritional supplementation, cognitive training, psychological intervention, etc. Thus, identification of frailty in non-dialysis dependent (NDD)-CKD patients may facilitate targeted interventions which may add to survival advantage and defer initiation of early dialysis.

The present study aimed to examine the association between CKD and frailty by evaluating the distribution of frailty among groups defined by different stages of the disease. The study also aimed to identify co-morbidities and associated health deficits among these subjects and their association with estimated glomerular filtration rate (eGFR) and urine albumin creatinine ratio (UACR). Furthermore, the study tried to ascertain previously unidentified confounders in this relationship.

## Methods

This cross-sectional study included ninety adult patients between 18–65 years of age who attended the out-patient department, the in-patient department, and the Nephrology Clinic of the Department of Medicine of UCMS and GTB Hospital, Delhi, with evidence of CKD between stages 1 to 4 after they provided a written and informed consent to participate. CKD was defined by serum creatinine based on the CKD Epidemiology Collaboration (CKD-EPI) equation as an estimated eGFR of <90 mL/min/1.73m^2^ or the presence of albuminuria (spot urinary albumin:creatinine ratio (UACR) of >30 mg/g)^7^. UACR was used as a marker for kidney function. Urinary albumin was estimated by nephelometry and urinary creatinine was estimated using Jaffe’s method^8^. UACR was calculated manually by finding the ratio of the two values.

Study subjects were divided into three groups of thirty participants each based on the eGFR:

- GROUP A: Stage 1 CKD + Stage 2 CKD
- GROUP B: Stage 3a CKD
- GROUP C: Stage 3b CKD + Stage 4 CKD

Subjects were evaluated for frailty and CKD, and details regarding their clinical and demographic features were recorded. There are several operational definitions of frailty that are usually rule-based such as the widely used phenotypic model developed by Fried et al.^9^ where a person is classified as frail if three or more symptoms are present. Counting deficits and summing the number of impairments in the cumulative deficits model is another way to define frailty as proposed by Searle et al.^10^ The phenotypic model and the cumulative deficits model show considerable overlap and statistical convergence^11^.

The *frailty* phenotype was determined using the Fried’s et al.^9^ criteria. Participants with three or more of the characteristics: weakness, slowness, unintentional loss of weight of 4.5 kg or more, exhaustion and low physical activity, were considered to be frail. In addition, health deficits were assessed using a pre-defined list of co-morbidities and deficits (appendix A) based on a method described by Searle et al.^10^ Deficits covered various aspects of health including co-morbidities, family composition, physical parameters, mental status, social profile, psychological profile, daily activities, and health-related complaints. Apart from CKD and hypertension, polypharmacy, BMI, tachycardia, grip strength, DM, hospitalization, smoking, vision problems and headache were the most common deficits found in our study.

Furthermore, the participants were evaluated for depression using the 4-point depression scale^12^. Those with scores of two or more were considered depressed. The *Get-up-and-go* test was performed and those who took more than 16 seconds to complete the test were considered to have failed the test^13^. The *Folstein Mini Mental State Examination (MMSE)* was performed and a memory was assessed with a total score of 30. Those who scored less than 24 (if literate) or 13 (if illiterate) were considered cognitively impaired^14^. The *World Health Organization Quality of Life Brief (WHOQOL-BREF)* questionnaire was administered to assess the quality of life of study participants and the transformed score for each domain was obtained^15^. A higher score denotes that the quality of life is good, whereas a lower score denotes that the quality of life is poor. The Barthel’s index of activities of daily living was used to evaluate physical function and dependence. The score ranges from 0–99, and a higher score indicates lower dependency. Blood and urine samples were collected for analysis of urine routine microscopy, hemoglobin, erythrocyte sedimentation rate (ESR), semi-quantitative C-reactive protein, blood sugar (fasting and post-prandial), kidney function test, liver function test, lipid profile, urine albumin-creatinine ratio, and eGFR.

The data was analyzed using Stata software (Version 13, Stata Inc, USA). Descriptive analysis was presented using means (standard deviations) and proportions (frequency and percentages). The difference in the proportion of frail subjects was compared between different groups. One-way analysis of variance, Chi-square test (Fisher’s exact test when applicable), and Student’s *t*-test were used to study the difference between groups, as applicable. The association between number of health deficits and eGFR and UACR was assessed using Spearman’s correlation coefficient. After univariate analysis, multiple logistic regression models were developed to explore the relationship between frailty and kidney disease and identify factors that may predict frailty after adjusting for chronic kidney disease.

The study was approved by the Institutional Ethics Committee for human research at our institution and participants were enrolled only after they signed an written informed consent.

## Results

### Demographic Parameters

A total of 90 participants were included in three groups (thirty each), stratified by the stage of CKD. The mean age of the participants was 49 years (±12.4). There were 51 (56.7%) women. The mean body mass index of the participants was 22.8 kg/m^2^ (±5.1). Of our subjects, 19 (21.1%) were frail. The baseline parameters of all the participants in the three groups are presented in Table 1.

**Table 1.** Baseline characteristics of the participants

Variable	Group A (N = 30)	Group B (N = 30)	Group C (N = 30)	Total (N = 90)	Significance
Age (years)	47.4 ± 13.4	51.6 ± 11.8	48.0 ± 12.0	49.0 ± 12.4	0.4
Male (%)	15 (50.0)	14 (46.67)	10 (33.33)	39 (43.33)	0.4
Married (%)	26 (86.67)	23 (76.67)	26 (86.67)	75 (83.33)	0.03*
BMI (kg/m^2^)	22.3 ± 4.1	23.2 ± 4.7	22.9 ± 6.3	22.8 ± 5.1	0.8
SBP (mmHg)	135.6 ± 22.5	143.3 ± 20.1	137.6 ± 22.3	138.8 ± 21.6	0.4
DBP (mmHg)	81.3 ± 11.5	85.0 ± 14.4	84.9 ± 11.0	83.7 ± 12.4	0.4
Get up and go time (s)	10.7 ± 3.3	11 ± 5.1	11.3 ± 4.0	11.0 ± 4.2	0.9
MMSE	26.9 ± 4.0	26.5 ± 3.6	25.8 ± 3.4	26.4 ± 3.7	0.5
Barthel’s ADL Score (0–20)	19.8 ± 0.8	19.9 ± 0.5	19.1 ± 2.5	19.6 ± 1.6	0.1
Pill burden	8.8 ± 3.8	11.1 ± 3.3	12.2 ± 4.0	10.7 ± 3.9	0.002*
Falls present (%)	5 (16.67)	5 (16.67)	4 (13.33)	14 (15.56)	0.9
Depression	4 (13.33)	6 (20.00)	4 (13.33)	14 (15.56)	0.6
Hemoglobin	12.1 ± 2.3	11.7 ± 1.8	10.8 ± 1.7	11.5 ± 2.0	0.03*
Blood urea (mg/dL)	30.0 ± 9.9	39.9 ± 13.1	60.8 ± 21.1	43.5 ± 20.0	–
Serum creatinine (mg/dL)	1.0 ± 0.2	1.4 ± 0.2	2.2 ± 0.5	1.5 ± 0.6	–
Serum protein (g/dL)	7.2 ± 1.1	7.1 ± 0.6	7.0 ± 0.8	7.1 ± 0.9	0.5
Serum cholesterol (mg/dL)	165.6 ± 52.7	164.1 ± 38.4	168.3 ± 99.7	166.0 ± 68.0	0.97
UACR (mg/g)	230.4 ± 316.6	193.2 ± 243.8	607.2 ± 1040.2	343.6 ± 663.3	–
eGFR (mL/min)	81.2 ± 16.6	50.7 ± 4.9	29.6 ± 8.0	53.8 ± 23.9	–
**Frail**	**3 (10.0)**	**4 (13.3)**	**12 (40.0)**	**19 (21.1)**	<0.01*
Weak handgrip	7 (23.3)	7 (23.3)	16 (53.3)	30 (33.3)	0.02*
Slow gait speed	3 (10.0)	4 (13.3)	7 (23.3)	14 (15.6)	0.3
Weight loss	3 (10.0)	1 (03.3)	9 (30.0)	13 (14.4)	<0.01*
Exhaustion	3 (10.0)	8 (26.7)	9 (30.0)	20 (22.2)	0.1
Low Activity	14 (46.7)	13 (43.3)	18 (60.0)	45 (50.0)	0.4
Count of deficits	4.5 (3, 7)	5 (4, 7)	4 (3, 9)	5 (3, 7)	0.96

Data are presented as mean (±standard deviation) or median (interquartile range) or number (percentage) as applicable. SBP: Systolic Blood Pressure; DBP: Diastolic Blood Pressure; ADL: Activities of Daily Living; MMSE: Mini Mental State Examination; QOL: Quality of Life; UACR: Urine-Albumin Creatinine Ratio; eGFR: estimated Glomerular Filtration Rate. *p < 0.05.

### Factors Affecting Frailty

Univariate analysis of the distribution of independent variables with frailty indicated associations with depression, performance on Barthel’s ADL index, and WHOQOL. Nine (47%) of the frail participants were depressed while only 11 (15.5%) of those who were not frail had depression. Frail individuals performed poorly on Barthel’s ADL (18.1 vs 20) and had a lower score on the WHOQOL scale. Frail subjects identified according to Fried et al.^9^ were not evenly distributed between groups; most of them – 12 (40%) – were in Group C (p < 0.01). The mean eGFR was significantly lower in frail subjects (44.2 ± 26.8 mL) compared to those who were not frail (56.4 + 22.6 mL; p = 0.047). At the same time, urine ACR was significantly higher in frail subjects (617.9 ± 1216) compared to those who were not frail (270.2 ± 392; p = 0.04). The univariate analysis of frailty is presented in detail in Table 2.

**Table 2.** Comparison between frail (fried’s phenotype) and non-frail participants

Variable	Non-frail (n = 71)	Frail (n = 19)	Total (n = 90)	P value
Age (years)	48.2 ± 12.9	52.2 ± 10.0	49.0 ± 12.4	0.2
Male sex (n, %)	29 (40.85)	10 (52.63)	39 (43.33)	0.357
Married (n, %)	59 (83.10)	16 (84.21)	75 (83.33)	0.633
BMI (kg/m^2^)	22.7 ± 4.6	23.2 ± 6.6	22.8 ± 5.1	0.7
SBP (mmHg)	138.3 ± 20.6	141.0 ± 25.5	138.8 ± 21.6	0.6
DBP (mmHg)	83.6 ± 12.3	84.2 ± 12.8	83.7 ± 12.4	0.9
Timed get up and go test (s)	10.1 ± 2.1	14.2 ± 7.3	11.0 ± 4.2	<0.001*
Barthel’s ADL Score (0–20)	20.0 ± 0.0	18.1 ± 3.1	19.6 ± 1.6	<0.001*
MMSE	26.6 ± 3.8	25.5 ± 3.2	26.4 ± 3.7	0.2
Pill burden	10.4 ± 3.9	11.6 ± 4.0	10.7 ± 3.9	0.3
History of falls (n, %)	9 (12.68)	5 (26.32)	14 (15.56)	0.145
Depressed (n, %)	10 (14.29)	9 (45.00)	19 (21.11)	0.003*
WHOQOL -BREF				
Physical domain	64.5	45.3	60.4 ± 18.0	<0.001*
Psychological domain	62.1	53.1	60.2 ± 9.3	<0.001*
Social domain	68.0	60.5	66.4 ± 14.5	0.046*
Environmental domain	64.7	54.3	62.5 ± 14.5	0.005*
Hemoglobin (g/dL)	11.6 ± 2.0	11.2 ± 1.9	11.5 ± 2.0	0.4
Blood urea (mg/dL)	42.1 ± 19.7	48.9 ± 20.7	43.5 ± 20.0	0.2
Serum creatinine (mg/dL)	1.4 ± 0.6	1.8 ± 0.7	1.5 ± 0.6	0.01*
Serum protein (g/dL)	7.2 ± 0.8	6.9 ± 1.0	7.1 ± 0.9	0.3
Serum cholesterol (mg/dL)	163.0 ± 45.2	177.3 ± 121.4	166.0 ± 68.0	0.4
UACR (mg/g)	270.2 ± 391.9	617.9 ± 1215.6	343.6 ± 663.3	0.04*
eGFR (mL/min/1.73m^2^)	56.4 ± 22.6	44.2 ± 26.8	53.8 ± 23.9	0.05*
Count of deficits	4 (3, 6)	7 (6, 16)	5 (3, 7)	<0.001*

Data are presented as mean (±standard deviation) or median (interquartile range) or number (percentage) as applicable. SBP: Systolic Blood Pressure; DBP: Diastolic Blood Pressure; ADL Activities of Daily Living; MMSE: Mini Mental State Examination; QOL: Quality of Life; UACR: Urine Albumin Creatinine Ratio; eGFR: estimated Glomerular Filtration Rate. *p < 0.05.

After adjusting for age, sex, depression, and cognitive impairment, patients in Group C were found to be 9 times more likely to be frail compared to those in Group A (OR = 8.9; p = 0.009) in multivariate logistic regression analysis. Those depressed were 6 times more likely to be frail after adjustment (OR = 6.3; p = 0.005). The results of the logistic regression analyses are presented in Table 3.

**Table 3.** Crude and adjusted odds ratios for frailty in logistic regression analyses

Variable	Crude OR (95% CI)	Adjusted OR (95% CI)	P value
Age	1.03 (0.98–1.08)	1.00 (0.94–1.06)	0.9
Sex	1.61 (0.58–4.45)	4.30 (0.94–19.65)	0.06
Depression	4.91 (1.62–14.84)	6.30 (1.72–23.10)	0.005
Cognitive impairment	0.92 (0.81–1.05)	0.85 (0.71–1.03)	0.09
CKD Group A (reference category)	1	1	
CKD Group B	1.38 (0.28–6.80)	1.47 (0.23–9.21)	0.7
CKD Group C	6.00 (1.48–24.30)	8.91 (1.74–45.66)	0.009

OR: Odds Ratio, CI: Confidence Ratio, CKD: Chronic Kidney Disease.

### Health Deficit Count and Frailty in CKD

**Figure 1.**
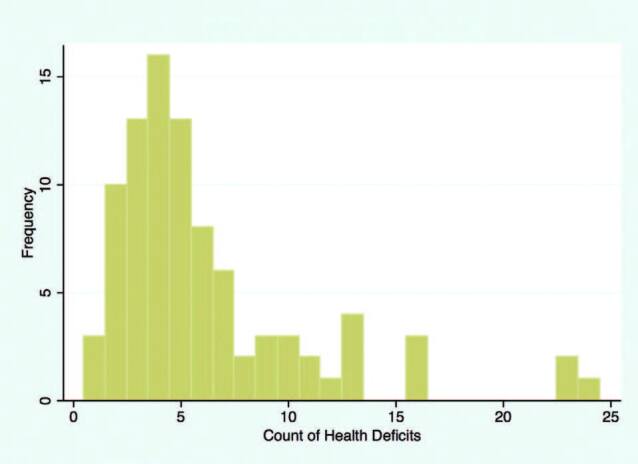
Health deficit counts in the participants.

A median count of five deficits (IQR; 3, 7) was observed in our study subjects. The maximum count of deficits was 24. A histogram of count of deficits is shown in Figure 1.

**Figure 2.**
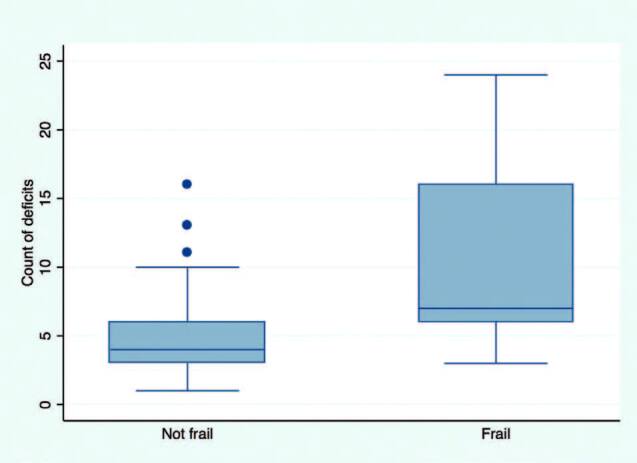
Health deficit counts by frailty status. The median counts were significantly different between frail and not frail participants (Fisher exact; p < 0.001).

**Figure 3.**
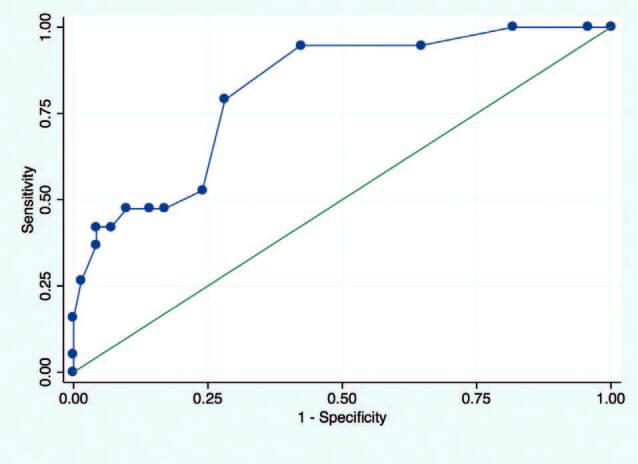
Receiver operator characteristics curve for frailty and deficit count. Area under the curve (AUC) = 0.8 (95% CI: 0.7, 0.9). A count greater than 6 was able to classify frail individuals with a sensitivity of 0.79 and a specificity of 0.72.

The median deficit count was 7 (IQR, 6, 16) in those identified as frail by Fried et al.^9^ phenotype index and 4 (IQR, 3, 6) in those classified as not frail. The median counts were significantly different between frail and non-frail participants (Fisher’s exact; p < 0.001). A box plot of deficit counts in frail and non-frail individuals is presented in Figure 2. A receiver operator characteristic curve analysis revealed an area under the curve (AUC) of 0.8 (95% CI: 0.7, 0.9). A count greater than 6 was able to characterize frail individuals with a sensitivity of 0.79 and a specificity of 0.72. The ROC graph is presented in Figure 3.

**Figure 4.**
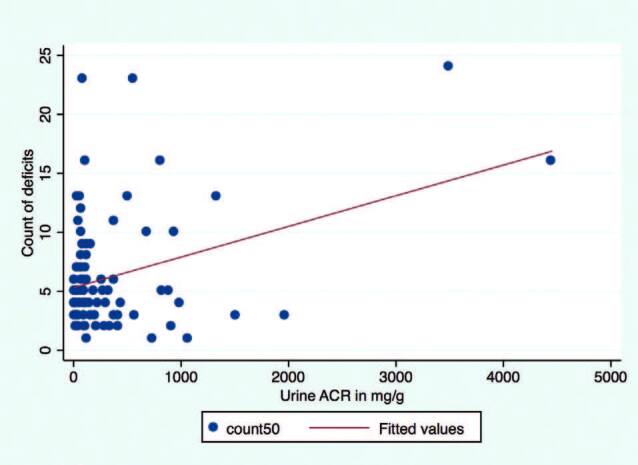
Health deficit counts and urine ACR. Scatter plot shows outliers. Urine ACR: urine albumin creatinine ratio (mg/g).

**Figure 5.**
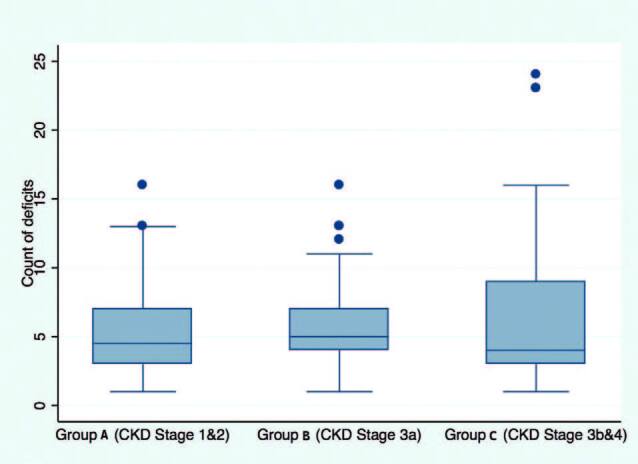
Health deficit counts by study groups of NDD-CKD. The median deficit count was not significantly different between the groups defined by CKD stage. NDD CKD: Non-dialysis-dependent chronic kidney disease; GROUP A: Stage 1 CKD + Stage 2 CKD; GROUP B: Stage 3a CKD; GROUP C: Stage 3b CKD + Stage 4 CKD.

The median deficit count was not significantly different between the groups defined by CKD stage. The count of deficits showed a significant correlation with UACR (r = 0.4; p < 0.001) but not with eGFR (p ≤ 0.4). However, the association between UACR and count of deficits could be biased due to the presence of outliers, as shown in Figure 4. A box plot of count of deficits is presented in Figure 5.

## Discussion

Of the 90 participants included in our study, 19 were identified as frail using the Fried’s phenotype index. Of those, three (10%) were in group A, four (13.3%) in group B, and twelve (40%) in group C, indicating an association between frailty and advancing kidney disease. The mean estimated GFR was lower (44.2 mL) and ACR was higher (617.9) in frail subjects compared to those who were not frail (56.4 mL and 280.2, respectively). Frail subjects were more likely to be depressed, had a higher count of health deficits, poorer performance on the Barthel’s ADL index, and lower scores on the WHOQOL.

### Frailty and CKD

The occurrence of frailty as defined by Fried’s criteria was 21.1% in our study. Mansur et al.^16^, in Brazil, conducted a study with 61 pre-dialysis CKD patients of stages 3-5 and found a 42.6% prevalence of frailty. Lee et al.^17^ in 2014 in Korea recruited 168 CKD subjects of stage 2-4 and found a 37.5% prevalence of frailty. These studies showed greater prevalence of frailty, which may be due to various factors. Firstly, the mean age of participants in our study was 49 years compared to 60.5 years in the Brazilian study and 62.5 years for women and 67.8 years for men in the Korean study. Secondly, none of these studies included stage 1 CKD subjects and both included more patients with higher CKD stages. Furthermore, the comparison between various studies may not be appropriate due to different frailty criteria used and varying characteristics of studied populations.

### Frailty and Health Deficits

A significant association between frailty and health deficits was found in our study. Hubbard et al.^18^ investigated whether CKD could be studied using frailty index and found a good correlation between CKD and modified frailty phenotype. Drost et al.^19^ in 2013 in the Netherlands studied 95 end-stage renal disease (ESRD) patients and found that broader definitions like frailty index provide higher prevalence compared to a physical assessment.

### Health Deficits, eGFR and UACR

Our study did not find significant associations between health deficits and eGFR and UACR. It is possible that the small difference in mean deficits that we observed could not reach statistical significance due to the small sample size. Also, deficits were assessed with equal weight, thereby underestimating some parameters and overestimating others, and this may have weakened the association between deficit count and eGFR and UACR. Similar to our study, Mansur et al.^16^ and Drost et al.^19^ also did not find any association between frailty and eGFR in their studies. Loaiza-Félix et al.^20^ in 2012-13 in Mexico compared a group of 35 type-2 diabetes mellitus patients with frailty to a control group of 35 frail patients without diabetes. They did not find any association of proteinuria with frailty in diabetic nephropathy patients.

### Frailty Components

Low physical activity was the most common deficit, present in 50% of participants, followed by weakness, which was present in 32.2% of participants. Roshanravan et al.^21^ found that the most common frailty components were inactivity (35.1%), exhaustion (31.8%), and slowness (25.9%). Delgado et al.^22^ studied 812 participants of the Modification of Diet in Renal Disease study and found that the most common frailty components were low physical activity (47%) and poor physical function (23%). Fried’s frailty phenotype, a construct based on individual component questions, may be complex to interpret in different cultural contexts and should be viewed in an appropriate perspective.

It has been observed earlier that loss of muscle mass is central to the development of the frailty syndrome. In CKD, handgrip strength has been recommended as a surrogate measure of protein-energy status and functional status^23^. The current research also found a significant association between CKD severity and handgrip strength. While the proportion of subjects with weak handgrip strength was 23.3% in CKD Group A and Group B, it was more than double (53.3%) in the CKD Group C that had the highest severity of CKD.

### Depression, Functional Ageing, Quality of Life and Frailty in CKD

We report an association between depression, QOL, ADL, and frailty. Bautovich et al.^24^ reviewed the prevalence, pathogenesis, associations, and management of depression in CKD and reported a prevalence of nearly 20%. John Sy et al.^25^ observed a significant association between depressive symptoms and frailty at baseline in a dialysis cohort, which was similar to what has been observed in the non-dialysis population (pooled OR 2.64, 95% CI 1.59–4.37 in meta-analysis). They reported that both frailty and depressive symptoms were independently associated with higher mortality compared to patients who were not frail and did not have depressive symptoms^26^.

The authors further noted an association between frailty and activities of daily living. In the Cardiovascular Health Study, 12% of individuals with kidney disease had impairment in activities of daily living compared with 7% of those without CKD^27,28^. In the NHANES study, 17% of adults above 65 years of age without CKD reported difficulty with ADL and 23% reported difficulty with instrumental ADL. The corresponding percentages for those with eGFR below 60 mL/min/1.73 m^2^ were 25% and 36%, respectively^5^. A significant association of frailty with all the four domains of WHOQOL was found, with physical domain having the strongest relation. Barros et al.^29^ used WHOQOL-BREF to assess QOL in 104 adult patients with ESRD and found that the physical domain was the most significant factor influencing QOL. Mansur et al.^16^ assessed QOL in Brazilian NDD CKD patients using SF-36. He observed that frailty correlated with all QOL domains except social domain. Lee et al. assessed QOL in Korean NDD CKD patients using SF-36 version 2 and found that frailty affected both mental and physical QOL in NDD CKD patients.

A greater proportion of our participants were functionally active, having been recruited from a hospital out-patient clinic, which requires a minimum ability to visit the hospital.

### Strengths and Limitations

Health deficits used in our study were designed specifically for an Indian population. The association of eGFR and UACR with health deficits has not been studied previously. To the best of our knowledge, this is the first study exploring the role of depression in the relationship between frailty and CKD. Our study did not include patients who receive regular dialysis due to advanced and end-stage renal disease and hence, is not representative of the entire spectrum of CKD. It would be interesting to study the progression of frailty with advancing renal disease. Our study includes equal number of cases in the three groups defined by CKD stage. This equality is not representative of the CKD prevalence in the community. Furthermore, various parameters and their cutoffs such as Fried’s frailty phenotype have been adopted from international definitions because no normative studies from India are available, but they may not be representative of our population.

## Conclusion

As many as 21% of subjects were found to be frail, among a total of 90 participants with CKD in our study. They were unevenly distributed with a skew indicating an association between frailty and advancing kidney disease. Frail individuals had a lower eGFR, higher ACR, were more likely to be depressed, had a higher number of health deficits, and poorer performance on Barthel’s ADL and WHOQOL.

The number of frail subjects increases in later stages of CKD, and there are associations with depression and poorer quality of life. Early identification of frailty in patients with CKD and further active screening for depression would allow informed decision making and optimal use of resources.

## Supplementary Material

The following online material is available for this article:

Appendix A
