# Supplementary material for: Frailty and chronic kidney disease: associations and implications
Source: J Bras Nefrol. 2023 Apr 17;45(4):401–9. doi: 10.1590/2175-8239-JBN-2022-0117en (PMC10726667; doi:10.1590/2175-8239-JBN-2022-0117en)
Supplement: Supplementary file 1 [file 2175-8239-jbn-2022-0117-s1.pdf]

Braz. J. Nephrol. (J. Bras. Nefrol.)
DOI: https://doi.org/10.1590/2175-8239-JBN-2022-0117en

Supplementary Material to "Frailty and chronic kidney disease: associations and implications"

Annexure A

The following health deficits were considered in the paper:

1) Based on clinical history

• Known hypertension

• Polypharmacy

• Diabetes Mellitus

• History of hospitalization in one year

• Smoking (bidi/cigarette)

• Difficulties in vision

• Feels everything is an effort

• Depression Scale (4 questions)

• Difficulty in hearing

• Sleep disturbances

• Feeling depressed/sad

• Coronary artery disease

• History of falls in last 6 months

• Tired all the time

• Decrease in food intake over last three months

• Gait imbalance

• Loneliness

• Dependence: needs help in grooming

• Stayed in bed at least half the day due to poor health in last one month

• Arthritis

• Impairment of memory

• Loss of more than 5 kg in one year

• Chronic obstructive pulmonary disease

• Dependence: needs help with bathing

• Dependence: needs help getting dressed

• Dependence: needs help walking around the house

• Dependence: needs help going up or down stairs

• Dependence: needs help with shopping

• Cerebrovascular accident /stroke

• Not living with family

• Dependence: needs help with using toilet

• Dependence: needs help getting in or out of chair

• Going out alone (does not go out alone)

• Difficulty in chewing food

• Dependence: needs help with eating

• Heart Failure

• Take medications (was previously able to take independently)

• Using phone (was previously able to do so independently)

• Living alone

2) Based on Clinical Examination

• Body mass index

• High blood pressure measurement

• Tachycardia

• Handgrip strength

• Time taken to walk 15 feet at normal speed

• Pallor

• Crepitations/wheeze in lung auscultation

• Mini Mental State Examination

• Tremor

3) Based on Lab Investigations

• Chronic kidney disease
